# Effect of violet and blue light activation on in-office bleaching with 6% hydrogen peroxide: color change, penetration into the pulp chamber and bleaching gel stability

**DOI:** 10.1007/s10103-026-04972-8

**Published:** 2026-07-29

**Authors:** Letícia Condolo, Michael W Favoreto, Laryssa Mylenna Madruga Barbosa, Isabela S Vardasca, Carlos Francci, Alessandra Reis, Alessandro D. Loguercio

**Affiliations:** 1 Ponta Grossa State University, Ponta Grossa, Brazil; 2 Universidade Tuiuti do Paraná, Curitiba, Brazil; 3 Universidade de São Paulo, São Paulo, Brazil

**Keywords:** Tooth bleaching, Bleaching agents, Hydrogen peroxide, Violet LED

## Abstract

This study evaluated color change, hydrogen peroxide (HP) penetration into the pulp chamber, gel properties, and temperature variation during in-office bleaching with 6% HP combined with different light sources. Sixty extracted sound premolars were randomly allocated into three groups (n = 20): (1) 6% HP without light activation, (2) 6% HP activated by violet light, and (3) 6% HP activated by blue light. Bleaching procedures followed the manufacturers’ recommendations. HP penetration into the pulp chamber was quantified using UV-Vis spectrophotometry. Color change was assessed with a digital spectrophotometer (WI_D_). Initial and final HP concentration and pH were determined by titration and a digital pH meter, respectively. Temperature variations during light activation were measured using a thermocouple. Data were analyzed using ANOVA and Tukey’s test (α = 0.05). The 6% HP + violet or blue light groups exhibited lower HP penetration compared with the 6% HP group (*p* = 0.02). The 6% HP + violet light group showed higher whitening values compared with the other groups (*p* < 0.001). The 6% HP group retained a higher HP concentration (*p* = 0.001). Light-irradiated groups exhibited higher temperature variation and greater pH reduction compared with the 6% HP group (*p* < 0.001). The violet-light protocol combined with 6% HP resulted in higher whitening values and reduced HP diffusion into the pulp chamber. However, light activation was also associated with increased temperature variation and slight pH reduction, which should be considered when interpreting the biological safety of these protocols.

## Introduction

Dental bleaching with hydrogen peroxide (HP) or carbamide peroxide is based on peroxide diffusion through enamel and dentin, where HP acts as a strong oxidizing agent that reacts with organic components of the dental substrate, altering its optical properties and producing a lighter appearance [1–5]. Although bleaching has traditionally been attributed mainly to chromophore oxidation, recent evidence suggests that HP-induced oxidation of organic components, particularly collagen, may also contribute to increased enamel and dentin opacity and light scattering [3–5]. Thus, chromophore oxidation remains an important and widely accepted mechanism, particularly for early and superficial color changes, while changes in the organic matrix may also contribute to the overall bleaching effect. Within this context, in-office dental bleaching can be performed with or without light activation, although the underlying oxidative process remains based on peroxide activity [1, 2]. 

Recently, the use of light irradiation combined with bleaching gels has gained popularity, although its clinical benefits remain controversial [6, 7]. Blue light has been suggested to enhance the activity of HP through photothermal effects and possible photochemical interactions [8, 9]. However, current evidence regarding its effectiveness remains inconsistent. Violet light has also been proposed as an alternative light source because of its potential interaction with HP and its possible influence on peroxide degradation and thermal behavior during bleaching procedures [10, 11]. Nevertheless, clinical studies have reported conflicting results regarding the efficacy of light-assisted bleaching protocols [11–16], and systematic reviews have consistently shown no significant improvement in bleaching efficacy when light-activated and non-activated protocols are compared [17–20]. 

Although the efficacy of light activation remains controversial, violet light (405–410 nanometers) has been proposed as an alternative adjunct to conventional in-office bleaching protocols. Similar to other light sources, violet light may generate a mild temperature increase during irradiation, which could influence pulpal responses. Its use has been investigated primarily because its short wavelength has been hypothesized to interact with superficial pigments and potentially enhance HP degradation through wavelength-dependent photochemical effects rather than through thermal mechanisms [10–12, 21, 22]. However, these mechanisms remain hypothetical, and the available experimental and clinical evidence is limited and inconsistent. Consequently, the exact role of violet light in enhancing bleaching efficacy has not yet been established and may depend on multiple factors, including the irradiation protocol and bleaching agent used.

High-concentration HP gels are commonly used in in-office bleaching because they produce faster results [1, 23], but they are often associated with adverse effects, particularly tooth sensitivity [24]. Sensitivity is the most frequently reported side effect, affecting many patients and strongly linked to the concentration of the bleaching agent [25, 26]. In light of these concerns, there is growing interest in developing safer and more tolerable alternatives, such as low-concentration HP gels [19, 27]. However, although low-concentration gels can achieve similar final whitening outcomes, they typically require longer application times, which has led to the exploration of light activation as a potential adjunct to accelerate bleaching performance.

Despite the growing number of studies on light-assisted bleaching, there is still a lack of consensus regarding whether alternative light wavelengths, particularly violet light, may behave differently when combined with low-concentration HP gels, since most previous evidence is based on conventional blue-light systems associated with medium- or high-concentration bleaching agents. Therefore, the present study was not designed to support the indiscriminate use of light-assisted bleaching, but to investigate the optical, physicochemical, and biological effects of different light wavelengths under low-concentration bleaching conditions. Most available studies have focused on high-concentration agents or have not simultaneously examined bleaching efficacy and biological safety parameters, such as HP diffusion and temperature variation. Within this context, evaluating these outcomes together may contribute to a better understanding of the potential benefits and limitations of alternative light-assisted bleaching protocols.

Therefore, this in vitro study primarily aimed to evaluate the effects of violet and blue light activation in combination with low-concentration HP (6%) on color change and HP penetration into the pulp chamber. The secondary objectives were to assess the physicochemical properties of the bleaching gel (initial and final HP concentrations and pH variation) and the temperature changes in human teeth treated with low-concentration HP, with and without light activation. Based on current scientific evidence, the null hypotheses tested were: (1) violet and blue light activation have no significant effect on bleaching outcomes compared to low-concentration HP gel without light activation; and (2) violet and blue light activation have no significant effect on HP diffusion into the pulp chamber compared to low-concentration HP gel without light activation.

## Methods and materials

### Ethical approval and tooth selection

This in vitro study was approved by the Research Ethics Committee of the State University of Ponta Grossa (PR/Brazil) under agreement number 7531728. Sixty caries-free maxillary first premolars were obtained from the tooth bank of the State University of Ponta Grossa (PR/Brazil). Each tooth was cleaned, disinfected in a 0.5% chloramine solution for 24 h, and stored in distilled water until use. To standardize the selection process, a 10x magnification microscope (Lambda LEB-3, ATTO Instruments, Hong Kong, China) was used to detect any morphological changes or enamel cracks.

### Inclusion and exclusion criteria

Teeth exhibiting any morphological changes or enamel cracks were excluded from the sample. Additionally, teeth with a Whiteness Index for Dentistry (WI_D_) greater than 20, as measured by a digital spectrophotometer (VITA Easyshade Advance 4.0, VITA Zahnfabrik, Bad Säckingen, Germany), were also excluded [28]. Teeth with buccal thicknesses below 2.5 millimeters or above 3.5 millimeters, as determined by radiography analyses (Timex 70 C, Gnatus, Ribeirão Preto, SP, Brazil) following the specimen preparation protocol, were excluded [29]. 

### Experimental groups

Sixty teeth were randomly assigned to three experimental groups (*n* = 20) to assess the effects of light activation on 6% HP (Whiteness HP Automixx 6%; FGM Dental Group, Joinville, SC, Brazil) bleaching. In all groups, the bleaching gel remained in contact with the tooth surface for 50 min per session over three sessions performed at seven-day intervals, regardless of the irradiation protocol used:

1. 6% HP Alone (Control Group): This group received only the 6% HP bleaching gel. No light activation was applied, so no irradiation cycles were performed.
2. 6% HP + Blue Light: In this group, the 6% HP bleaching gel was applied to the teeth and activated using a blue light source. The device used (Whitening Lase White Plus, DMC, São Carlos, SP, Brazil) emitted light at a wavelength of 450 ± 10 nanometers, combined with infrared radiation at 808 ± 10 nm. The irradiation protocol consisted of three cycles of 3 min each, with 15-minute intervals between cycles, totaling 9 min of irradiation per session, strictly according to the manufacturer’s recommendations for this device.
3. 6% HP + Violet Light: In this group, the 6% HP bleaching gel was applied to the teeth and activated using a violet light source. The device used (Bright Max Whitening, MMOptics, São Carlos, SP, Brazil) emitted light within the 405–410 nanometers wavelength range. The irradiation protocol consisted of twenty cycles of 1 min each, with 30-second intervals between cycles, totaling 20 min of irradiation per session, strictly according to the manufacturer’s recommendations for this device.

### Sample size calculation

In this in vitro study, we aimed to detect a clinically perceptible difference of approximately 2.6 ΔWI_D_ units between the group with no light activation and the groups with light activation [30]. Using the same bleaching gel as in the present investigation, a mean color change of 3.9 ± 2.7 ΔWI_D_ units was reported after two 50-minute sessions without light activation [31]. Considering a statistical power of 80% and a significance level of 5%, the sample size calculation indicated a minimum requirement of 17 teeth per group. To compensate for potential specimen loss during the experimental procedures, three additional teeth were included, resulting in a total of 20 teeth per group.

### Specimen preparation and randomization

A low-speed diamond disk (Isomet 1000, Buehler Ltd, Lake Bluff, IL, USA) was used to section each tooth root approximately 3 millimeters apical to the cementoenamel junction. The pulp tissue was carefully removed and rinsed with deionized water [32]. To further expand access to the pulp chamber, a spherical bur (#1014, KG Sorensen, SP, Brazil) was utilized to create an opening capable of holding up to 25 µL of solution (LABMATE Soft, HTL Lab Solutions, Warsaw, Poland).

Radiographic images were then taken using the Timex 70 C X-ray machine (Gnatus, Ribeirão Preto, SP, Brazil), with each specimen positioned mesially or distally against the X-ray film. Radiographs were captured at a 0.5-second exposure time and a 30-centimeter focus-to-object distance (70 kVp, 7 mA), with the central beam directed at a 90° angle to the tooth’s distal surface. After exposure, the images were digitally processed, and buccal tooth thickness was measured using New IDA software (Dabi Atlante, Ribeirão Preto, SP, Brazil) [32]. 

Each specimen received a unique identification code before randomization. To ensure balanced baseline WI_D_ values across the experimental groups, specimens were stratified according to their initial WI_D_ values and then randomly assigned within each stratum using a random sequence generated in Microsoft Excel. The randomization sequence was generated by an independent researcher who was not involved in the experimental procedure, thereby ensuring allocation concealment until group assignment. Due to the nature of the bleaching protocols, operator blinding during the interventions was not feasible. However, the evaluator responsible for outcome assessment remained blinded to the experimental group assignments throughout the study.

### Initial color measurements

To standardize spectrophotometer positioning, individual impressions were created for each specimen using a dense green silicone [33] paste (Coltoflax and Perfil Cub Kit, Vigodent, Rio de Janeiro, RJ, Brazil). A 6-millimeter-diameter window was then cut to expose the buccal surface in the middle third of each specimen, using a standardized circular punch to ensure precision [32]. 

Initial color coordinates (L*, a*, and b*) were measured with a digital spectrophotometer (VITA Easyshade Advance 4.0, VITA Zahnfabrik). Before each measurement, the spectrophotometer was calibrated and positioned within the designated measurement window. To minimize dehydration effects, the specimens were maintained in a moist environment until evaluation. Excess surface moisture was gently removed with gauze immediately before measurement, and the color assessment was performed without prolonged air exposure. The L* value indicates lightness on a scale from 0 (black) to 100 (white); the a* value represents color on the red-green axis; and the b* value represents color on the yellow-blue axis. Each reading was displayed on the device screen. To assess baseline Whiteness Index for Dentistry (WI_D_) values, the following formula was applied: WI_D_ = 0.551 × L – 2.324 × a – 1.1 × b [34]. As previously described, only teeth with a WI_D_ lower than 20 were included in the study [28]. 

### Obtaining the study calibration curve

The analytical reagents used in this study were not pre-purified, and all solutions were prepared using deionized water. To begin, a standard reference line was generated from a stock solution of 5,000 µg mL^-1^, which was made by diluting a concentrated HP solution (HP 35%, Pharmacy Eficácia, Ponta Grossa, PR, Brazil). This stock solution was further diluted in an acetate buffer (pH = 4) and calibrated following conventional methods. To verify the analytical grade and actual concentration, titration was performed using a potassium permanganate solution [29, 32]. 

After confirming the initial concentration, a series of volumetric dilutions ranging from 0.000 to 0.414 µg mL^-1^ were prepared to create the calibration curve. Known concentrations of HP were then placed in glass tubes and analyzed in a Cary 100 UV-Vis spectrophotometer (Varian, Palo Alto, CA, USA). This process established a standard reference line that was subsequently used to extrapolate results for the study samples (*R* = 0.998; data not shown) [32]. 

### Treatment protocols and HP penetration into the pulp chamber

For all groups, the specimens were secured vertically to a wax plate with the occlusal surface facing downward. Before applying the bleaching agent, a light-cured resin barrier was placed on the buccal surface of each specimen to isolate an area of 6 mm x 6 mm (Topdam, FGM Dental Products, Joinville, SC, Brazil) for gel application and to prevent gel overflow to adjacent tooth surfaces. The curing time for each specimen was 20 s with 1000 mW/cm^2^ (Quazar, FGM Dental Products, Joinville, SC, Brazil). To retain any HP that might enter the pulp chamber during bleaching, a 25-µL aliquot of acetate buffer was added to the pulp chamber of each specimen.

A single experienced operator applied all materials. The bleaching agent was applied to the buccal enamel surface according to the protocols of the experimental groups, ensuring complete coverage of the target surface. For the light-activated groups, the bleaching gel was applied to the buccal enamel surface according to the irradiation protocols previously described for each device, while maintaining a 2-cm distance between the light source and the specimen throughout irradiation.

The blue-light device, Whitening Lase White Plus, emitted light at 450 ± 10 nm combined with infrared radiation at 808 ± 10 nm. Each LED diode produced 350 mW, resulting in a total optical power of 1.2 W and an irradiance of 140.2 mW/cm². The violet-light device, the Bright Max Whitening, emitted light within the 405–410 nm range; each LED diode produced 350 mW, resulting in the same optical power of 1.2 W and irradiance of 140.2 mW/cm². However, due to differences in irradiation time between the protocols, the total delivered energy density differed between the light sources, corresponding to approximately 75.7 J/cm² for blue light and 168.2 J/cm² for violet light.

After 50 min per session, the bleaching gel was removed with gauze and the area was rinsed thoroughly with deionized water. This procedure was repeated three times at seven-day intervals following the same application protocol. Between sessions, the specimens were stored in artificial saliva [35] (Pharmacy Eficácia, Ponta Grossa, PR, Brazil) containing carboxymethylcellulose, sodium chloride, potassium chloride, magnesium chloride, dibasic calcium phosphate, glycerin, xylitol, and distilled water. The artificial saliva was refreshed daily and kept at a constant temperature of 37 °C.

Next, the acetate buffer solution was collected from the pulp chamber using a mechanical micropipette after four rinses with 25 µL of acetate buffer, and then transferred to a glass tube. Subsequently, distilled water, Leucocrystal Violet solution (0.5 mg mL⁻¹; Sigma Chemical Co., St. Louis, MO, USA), and horseradish peroxidase enzyme solution (1 mg mL⁻¹; Peroxidase Type VIA, Sigma Chemical Co.) were added. The resulting violet-colored solution exhibited peak absorbance at 591 nm, which was measured using a Cary 100 UV-Vis spectrophotometer (Varian, Palo Alto, CA, USA). HP concentration (µg mL⁻¹) was determined from absorbance values using a previously established calibration curve.

### Color change evaluation

After the bleaching phase, the final color coordinates (L*, a*, and b*) were recorded exactly as described in the section *Initial color measurements*. To assess color changes, measurements were taken at baseline and again at one week, two weeks, three weeks, and one month after bleaching. The color change between these points was calculated with the WI_D_ formula, as previously described. The WI_D_ was selected because it is considered a more specific and clinically relevant parameter for evaluating tooth-whitening outcomes, showing a strong correlation with perceived dental whiteness in bleaching studies. Perceptual changes were considered clinically noticeable when initial and post-bleaching color differences when the WI_D_ exceeded 2.6 [30, 36]. A 50:50 acceptability threshold was used, considering values below this threshold to be clinically insignificant [30, 36]. 

### Initial and final concentrations of bleaching agents

The bleaching gels used in this study were titrated with a standardized potassium permanganate solution both before and after the bleaching procedure, as described in previous research [28, 32]. This titration was conducted to determine the initial and final concentrations of the bleaching gel. Each analysis was performed in triplicate to ensure accuracy and reliability. Variations within a range of -30% to + 10% from the manufacturer’s stated concentration were considered acceptable.

### pH measurements of bleaching agents

The pH of each bleaching agent was measured using a pH meter (Extech pH100, Extech Instruments, Nashua, NH, USA), applied directly to the bleaching gel on the tooth surface [37, 38]. Readings were taken at multiple intervals, starting immediately after gel application and then every 10 min thereafter. To ensure accuracy and reliability, each measurement was performed in triplicate at each time point.

### Temperature analysis

In this analysis, the same samples used in previous experiments were fitted with ultra-fast response T-type thermocouples (0.011″ diameter, IT-23 Physitemp Instruments, Clifton, NJ, USA), which were inserted into the buccal pulp horn (dentin), as described in prior studies [22, 39–44]. Temperature variations were monitored throughout the second bleaching session. To enhance heat conduction and ensure stable contact between the thermocouple tip and the pulp horn, thermal paste (Implastec, Votorantim Ind. Brasileira, São Paulo, SP, Brazil) and wax (Lysanda, São Paulo, SP, Brazil) were applied.

### Statistical analysis

The data were analyzed statistically, starting with the Kolmogorov-Smirnov test to assess normality and the Bartlett test to assess homogeneity of variances (data not shown). The buccal thickness (mm), HP concentration in the pulp chamber (µg mL^-1^), and temperature change (ΔT, ^o^C) values were analyzed by a one-way ANOVA (treatment). For the analysis of color change (WI_D_), pH variation, and HP concentrations of the gel (%), a two-way repeated measures ANOVA (treatment vs. time) was applied. Sphericity assumptions were assessed using Mauchly’s test, and when violated, Greenhouse–Geisser corrections were applied. For post-hoc comparisons, a Tukey’s test was used (α = 0.05). All tests were performed using the Statistica for Windows software (StatSoft, Tulsa, OK, USA).

## Results

### Color change

Table 1 presents the mean values and standard deviations of the WI_D_ for the different experimental groups over time. A significant interaction effect between treatment and evaluation time was observed (*p* < 0.001; η²*p* = 0.175), as well as significant effects for time (*p* < 0.001; η²*p* = 0.651) and treatment (*p* < 0.001; η²*p* = 0.398).

**Table 1:** Means and standard deviations of the Whiteness Index for Dentistry (WI_D_) of the different experimental conditions across several time assessments

Parameters	Time assessments	6% HP	6% HP + blue light	6% HP + violet light
WI_D_	Baseline	16.4 ± 2.5 F	16.7 ± 2.0 F	16.6 ± 2.3 F
	1 week	20.1 ± 2.1 E	22.3 ± 2.1 D	26.8 ± 2.2 B
	2 week	21.9 ± 2.4 D, E	23.9 ± 2.9 D, C	28.0 ± 2.1 A, B
	3 week	23.2 ± 2.5 D, C	24.0 ± 2.5 C	28.4 ± 2.9 A
	1 month	23.6 ± 2.5 D, C	25.9 ± 2.8 B	28.7 ± 3.0 A

(*) Comparisons are valid for different time assessments within the same color change parameter. Identical capital letters indicate statistically similar means (Tukey’s test; *p* > 0.05)

At baseline, no significant differences were observed among the groups (*p* = 0.98), indicating similar initial tooth color. All groups exhibited progressive increases in WI_D_ values over time, although the magnitude of whitening varied according to treatment and evaluation time. Whitening values increased significantly after 1 week compared with baseline and after 3 weeks compared with both baseline and 1-week evaluations for all groups (*p* < 0.001).

The 6% HP + violet light group exhibited higher WI_D_ values than the other groups at all post-bleaching evaluations (*p* < 0.001). At 1 month, the estimated mean WI_D_ values for the 6% HP, 6% HP + blue light, and 6% HP + violet light groups were 23.0, 25.2, and 29.0, respectively (95% CI: 21.0–25.0; 23.2–27.2; and 27.0–31.0). In contrast, the 6% HP alone and 6% HP + blue light groups showed similar WI_D_ values at most evaluation periods, except after 1 week and 1 month.

The between-group ΔWI_D_ differences involving the violet-light protocol exceeded the reported perceptibility (ΔWI_D_ = 0.7) and, when compared with the 6% HP group, the acceptability thresholds (ΔWI_D_ = 2.9). These findings indicate that the additional whitening achieved with the violet-light protocol would be perceptible to participants and, relative to the non-activated protocol, would also be considered clinically acceptable.

### HP Concentration in the pulp chamber

Table 2 presents the buccal tooth thickness measurements and HP concentrations detected in the pulp chamber. Buccal thickness did not differ among the groups (*p* = 0.82). HP diffusion into the pulp chamber differed significantly among the experimental groups (*p* = 0.01; η²*p* = 0.266), with higher HP diffusion into the pulp chamber observed for the 6% HP group compared with both light-activated groups (*p* = 0.02).

**Table 2:** Means and standard deviations of buccal thickness (mm) and the hydrogen peroxide (HP) concentration (µg mL^− 1^) detected within the pulp chamber in different experimental groups (*)

Experimental groups	Buccal thickness (mm)	HP concentration (µg/mL^− 1^)
6% HP	3.4 ± 0.2 A	0.038 ± 0.008 a
6% HP + blue light	3.3 ± 0.2 A	0.026 ± 0.014 b
6% HP + violet light	3.3 ± 0.2 A	0.022 ± 0.014 b

(*) Identical capital or lower-case letters indicate statistically similar means for each column (Tukey’s test; *p* > 0.05)

 No differences were observed between the light-activated groups (*p* = 0.89).

Table 3 presents the initial and final HP concentrations of the bleaching gel. A significant interaction effect between treatment and time was observed (*p* = 0.001). All groups showed reductions in HP concentration after treatment; however, the final HP concentrations differed among the groups. The 6% HP alone group retained the highest residual concentration, which was significantly higher than that observed for the 6% HP + violet light group (*p* = 0.001). Intermediate values were observed for the 6% HP + blue light and 6% HP + violet light groups showed similar residual HP concentrations (*p* > 0.05).

**Table 3:** Means and standard deviations of hydrogen peroxide concentrations (%) before and after the bleaching procedure, in the different experimental groups (*)

Experimental groups	Concentration of HP (%)	
	Initial	Final
6% HP	5.7 ± 0.0 A	5.2 ± 0.10 B
6% HP + blue light	5.7 ± 0.0 A	4.3 ± 0.09 C
6% HP + violet light	5.7 ± 0.0 A	4.2 ± 0.12 C

(*) Identical capital letter is statistically similar for each experimental groups (Tukey’s test; *p* > 0.05)

### pH Measurements of bleaching agents

**Fig. 1:**
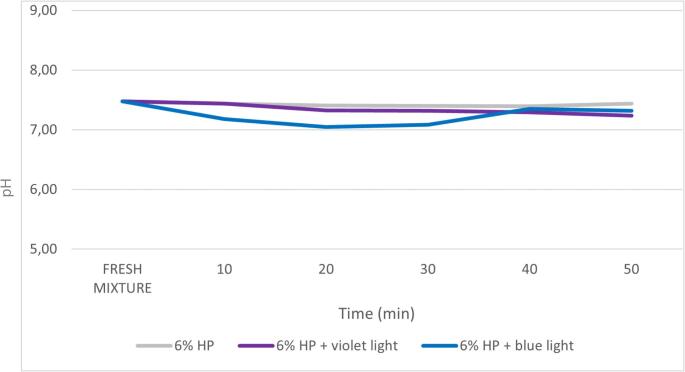
pH variation of 6% hydrogen peroxide solutions over time under different experimental conditions. Gray line: 6% HP alone; violet line: 6% HP + violet light; blue line: 6% HP + blue light

Figure 1 illustrates the pH variation over time for the different experimental conditions. A significant interaction effect between treatment and time was observed (*p* < 0.001). All groups exhibited similar initial pH values (7.5). Over the 50-minute evaluation period, distinct pH behaviors were observed among the groups (*p* < 0.001). While the 6% HP alone group maintained stable pH values, both light-activated groups showed pH reductions over time. The 6% HP + blue light group reached the lowest pH values at approximately 20 min, whereas the 6% HP + violet light group exhibited a more gradual decrease, reaching the lowest values at 50 min.

### Temperature analysis

Table 4 displays the temperature variations across the different experimental groups. A significant interaction effect between treatment and time was observed (*p* < 0.001). Temperature increased in all groups, although the magnitude of change differed among treatments. The 6% HP alone group exhibited the lowest temperature variation (0.30 °C), whereas both light-activated groups showed higher temperature increases (*p* = 0.001), reaching 8.82 °C for blue light and 7.95 °C for violet light, with no differences between them (*p* > 0.05).

**Table 4:** Means and standard deviations of temperature change (ΔT, ^o^ C), in the different experimental groups (*)

Experimental groups	ΔT (^o^ C)
6% HP	0.30 ± 0.30 A
6% HP + blue light	8.82 ± 0.72 B
6% HP + violet light	7.95 ± 0.60 B

(*) Identical capital letter is statistically similar for each experimental groups (Tukey’s test; *p* > 0.05)

## Discussion

The role of light activation in enhancing the efficacy of HP–based bleaching remains a topic of ongoing debate. Light sources have been hypothesized to accelerate the decomposition of HP into reactive oxygen species through photothermal and photochemical processes, which may contribute to enhanced bleaching efficacy [8, 45]. However, these mechanisms were not directly investigated in the present study. Previous systematic reviews have generally reported limited or no additional benefit when conventional light systems, such as blue light, are combined with HP gels [6, 17]. In the present study, the violet-light protocol resulted in higher whitening values throughout the evaluation period, whereas the blue-light protocol showed bleaching outcomes largely comparable to those of the non-activated protocol at most evaluation times. Therefore, the first null hypothesis was rejected.

One possible explanation for the differences between the present findings and those reported in previous systematic reviews is the methodological heterogeneity among studies, including HP concentration, wavelength characteristics, irradiation protocols, exposure time, total energy density, and outcome assessment methods [11–20]. Most of the studies included in these reviews evaluated conventional blue-light systems combined with medium- or high-concentration bleaching gels, whereas the present study investigated a violet-light protocol associated with low-concentration HP [17–22, 27]. In this context, it may be hypothesized that violet-light irradiation could have a greater influence when lower peroxide concentrations are used, since these protocols generate fewer reactive species than conventional high-concentration bleaching gels [8–10, 19, 21, 22, 46]. Nevertheless, this possible explanation remains speculative and requires confirmation in studies specifically designed to investigate the photochemical interactions involved. Furthermore, variations in irradiation parameters and the lack of protocol standardization among light-assisted bleaching studies may also contribute to the inconsistent findings reported in the literature [11–20]. 

Violet light (405–410 nm) has emerged as a newer technology that, due to its shorter wavelength and proximity to the absorption peak of chromophore molecules, may interact more directly with pigmented compounds on the enamel surface [46–48]. Although chromophore oxidation was not considered the primary mechanism underlying dental bleaching, this selective photoreactive interaction may contribute to early superficial color changes and enhance the initial bleaching response. Based on previous literature, this mechanism may promote degradation of superficial pigments through selective light absorption, potentially influencing enamel optical properties and contributing to a whiter appearance [46–48]. Unlike blue light, which is more commonly associated with photothermal effects [39, 40], violet light has been hypothesized to promote greater photochemical activity [46], which may explain the enhanced whitening outcomes observed in the present study.

Nevertheless, its ability to enhance whitening appears to be more evident when used in conjunction with low-concentration HP protocols, as suggested by recent studies [49, 50]. Previous investigations evaluating violet light associated with high-concentration bleaching gels (35–37% HP or carbamide peroxide) reported limited or no substantial additional whitening benefit compared with non-activated protocols, possibly because the high availability of reactive species already promotes intense bleaching activity [49, 50]. In contrast, studies using lower peroxide concentrations have suggested that violet light may enhance bleaching performance and accelerate early whitening outcomes, particularly during the initial treatment sessions [49, 50]. The additional action provided by violet light in low-concentration protocols may compensate for the lower release of reactive species, potentially improving bleaching efficacy even after the first session. This advantage is especially relevant in clinical contexts where earlier whitening stabilization is desired without increasing the HP concentration.

It is noteworthy that the color change pattern differed between the light-activated groups throughout the evaluation period. The specimens treated with violet light reached a plateau in WI_D_ values as early as the second week, while the blue-light group showed a more gradual increase up to one month. These findings suggest that violet light influences the early stages of bleaching differently from blue light, possibly through enhanced interaction with superficial pigmented compounds during the initial sessions [21]. One possible explanation, based on previous studies, is that the shorter wavelength and higher photoreactivity of violet light may contribute to earlier clinically perceptible whitening [21]. However, the present study did not directly evaluate the underlying photochemical mechanisms involved. In contrast, the blue light, which operates at a longer wavelength and induces greater thermal effects, showed a more gradual whitening pattern over time. However, its bleaching performance remained largely comparable to the non-activated group in most evaluations [51]. These distinct whitening patterns may indicate different interactions between the light sources and the bleaching gel. In the present study, violet light was associated with earlier whitening effects, whereas the blue-light group showed a more gradual response that remained largely comparable to the non-activated protocol. Similar bleaching kinetics have been previously reported [52]. 

It should be emphasized that the irradiation protocols differed substantially between the blue-light and violet-light devices, with total exposure times of 9 and 20 min, respectively, because the manufacturers’ instructions for each device were strictly followed. Therefore, the enhanced bleaching efficacy observed with violet light cannot be attributed exclusively to wavelength-related effects. The longer irradiation time may also have contributed to greater HP degradation, prolonged photochemical interaction, and increased cumulative energy delivery to the bleaching gel and dental substrate. Likewise, exposure time may have influenced the thermal behavior observed during bleaching, since longer irradiation periods may increase heat accumulation within the dental structure. Consequently, the distinct irradiation protocols represent a methodological limitation and a potential confounding factor when directly comparing the effects of the different light sources.

The irradiation protocols used in the present study are consistent with the variability previously reported in light-assisted bleaching investigations. Previous studies evaluating violet light have also employed fractionated irradiation protocols with repeated light exposure cycles during bleaching procedures [11, 49]. In addition, different irradiation modes, such as continuous and fractionated violet LED protocols, have been shown to influence both thermal behavior and bleaching outcomes [39]. In contrast, studies evaluating blue-light systems have frequently used shorter irradiation times and different energy-delivery approaches, particularly when infrared components are associated with the device [8, 12, 52]. This lack of protocol standardization makes direct comparisons among studies difficult and may partially explain the inconsistent findings reported in the literature regarding the effectiveness and safety of light activation during bleaching procedures.

The lower diffusion of HP in the light-irradiated groups may be associated with the potential of the light-activated bleaching process to accelerate peroxide degradation at the enamel surface, thereby reducing the amount of intact HP available to diffuse into deeper dental tissues, as suggested by previous studies [29]. These findings led to the rejection of the second null hypothesis. This interpretation is consistent with the lower final HP concentrations observed after titration in the light-activated groups, which may suggest faster breakdown of the active agent near the surface. Additionally, accelerated degradation may shorten the time window during which diffusion can occur, potentially limiting pulp chamber exposure [53, 54]. However, the present study did not directly evaluate peroxide degradation mechanisms or diffusion kinetics during irradiation. Therefore, these interpretations should be considered speculative and interpreted with caution. Although statistically significant differences in HP diffusion were observed among the groups, the absolute concentrations detected in the pulp chamber were very low under all experimental conditions. Therefore, the biological relevance of these differences remains uncertain. Furthermore, because pulpal response and tooth sensitivity were not evaluated in the present study, it is not possible to determine whether the reduced peroxide diffusion observed in the light-activated groups would translate into clinically meaningful biological benefits. Therefore, any association between lower hydrogen peroxide diffusion and a potential reduction in tooth sensitivity remains hypothetical and should be confirmed by well-designed clinical studies.

In low-concentration HP protocols, light activation could potentially contribute to a lower biological risk. Also, clinical studies indicate that the use of light activation, regardless of the type of light source, does not significantly alter the incidence or intensity of tooth sensitivity [16, 20]. Further clinical investigations are required to better understand the relationship between peroxide diffusion, light activation, and patient-reported outcomes in low-concentration HP protocols.

The overall pH variation among the light-activated groups was relatively small, ranging from approximately 7.5 to just above 7, remaining within a neutral to slightly alkaline range. Although light activation may influence HP diffusion and degradation dynamics, the extent of pH change observed in the present study was limited and remained within values commonly considered compatible with bleaching gel stability [29, 37, 38]. Therefore, under the experimental conditions evaluated, these variations are unlikely to substantially influence peroxide behavior or the overall physicochemical properties of the bleaching gel.

Regarding temperature analysis, both violet and blue light activation resulted in a significant temperature increase compared with the group treated with 6% HP alone, reaching values up to 8 °C above baseline. Notably, the temperature rise was similar between the two light-activated groups despite differences in wavelength and emission spectrum. These findings suggest that the thermal effects were not solely dependent on the light spectrum, but were also influenced by factors such as irradiance, exposure time, and the interaction between light, bleaching gel, and dental tissues [22, 39, 40, 43]. In particular, the longer exposure time used for violet light likely contributed to cumulative heat generation during the bleaching procedure. Although both devices operated with the same optical power (1.2 W) and irradiance, the violet-light protocol involved a longer irradiation time, resulting in greater cumulative energy delivery. Despite this difference, both light-activated groups produced comparable thermal outcomes, reinforcing previous evidence that overall energy absorption by the bleaching gel and dental structure may play an important role in temperature increase [43]. Light absorption by the gel and surrounding tissues likely promotes energy conversion into heat [55, 56], thereby increasing the temperature within the tooth structure.

Importantly, the temperature increases observed in the light-activated groups exceeded the commonly cited 5.5 °C threshold, which has historically been used as a reference for potential pulpal injury [44, 57]. However, the clinical applicability of this threshold has been debated because the original experimental model does not fully reproduce physiological pulpal conditions, including pulpal blood flow and heat dissipation mechanisms. Therefore, although the observed temperature increases warrant attention, they should not be interpreted as direct evidence of pulpal injury or clinical risk. Instead, these findings should be interpreted with caution and confirmed by studies conducted under clinically relevant conditions.

In the present study, violet light combined with low-concentration HP improved bleaching efficacy and reduced HP diffusion into the pulp chamber. However, these potential benefits should be interpreted cautiously, since the enhanced whitening performance was accompanied by substantial temperature increases during irradiation. Therefore, the potential thermal risks associated with light activation should be carefully weighed against the relatively limited additional whitening benefits reported for these protocols.

Furthermore, prolonged exposure times and greater cumulative energy delivery may increase thermal stress within the dental structure, particularly when high-intensity light sources are used. Consequently, irradiation parameters, including wavelength, exposure time, and energy density, should be carefully considered when evaluating the safety of light-assisted bleaching approaches. Accordingly, direct clinical extrapolation of the present findings remains limited. Further in vitro and clinical studies are necessary to determine whether physiological pulpal heat dissipation mechanisms are sufficient to prevent adverse biological effects during clinical light-assisted bleaching procedures.

As a limitation of this in vitro study, intrapulpal pressure, dentinal fluid movement, and pulpal blood flow were not simulated. Consequently, the HP diffusion values observed in the present study may overestimate the amount of peroxide reaching the pulp tissue under clinical conditions, where these physiological mechanisms may reduce peroxide penetration. Likewise, the thermal analysis may overestimate in vivo temperature changes because physiological heat dissipation was absent. Additionally, only a single HP concentration and two light-assisted bleaching protocols were evaluated, which may limit the generalizability of these findings. We also acknowledge that chloramine storage may alter the organic components of enamel and dentin. Furthermore, because the irradiation protocols followed the manufacturers’ recommendations, the light-assisted bleaching protocols differed substantially in exposure time and cumulative energy density. This represents an additional methodological limitation and a potential confounding factor that may have influenced the outcomes independently of wavelength, requiring caution when directly comparing the effects of the different light sources.

Finally, the blue-light device also included an infrared component, which may have contributed to the greater temperature increase and accelerated HP decomposition. Future in vitro and in vivo studies should investigate standardized irradiation protocols that isolate wavelength while controlling for exposure time and energy density, thereby improving our understanding of the relationships among bleaching efficacy, peroxide diffusion, thermal effects, and patient safety. Therefore, the present findings should be interpreted as a comparison between two distinct light-assisted bleaching protocols rather than as an isolated comparison of wavelength effects. Despite these methodological limitations, the present findings contribute to the current understanding of light-assisted bleaching by demonstrating that different clinical protocols may produce distinct physicochemical and thermal responses when associated with low-concentration HP protocols.

## Conclusion

Within the limitations of this in vitro study, the violet light-assisted protocol combined with 6% HP resulted in higher whitening than the non-activated protocol, whereas the blue light-assisted protocol showed bleaching results largely comparable to those of the non-activated group. However, this enhanced whitening effect cannot be attributed solely to wavelength because irradiation time, total energy density, and the presence of an infrared component differed between the protocols. In addition, both violet- and blue-light activation reduced HP diffusion into the pulp chamber compared with the non-activated protocol but produced temperature increases ranging from 7.9 to 8.8 °C, exceeding the commonly cited pulpal safety threshold. Although the biological significance of these temperature increases remains uncertain, they should be considered when interpreting the potential clinical safety of light-assisted bleaching protocols. Therefore, the present findings should be interpreted within the context of the evaluated clinical protocols rather than as isolated wavelength-dependent effects.

## Data Availability

All data supporting the findings of this study are available within the paper and its Supplementary Information.
